# Diagnosing myeloma in general practice: how might earlier diagnosis be achieved?

**DOI:** 10.3399/bjgp22X720737

**Published:** 2022-10-30

**Authors:** Lesley Smith, Jonathan Carmichael, Gordon Cook, Bethany Shinkins, Richard D Neal

**Affiliations:** Leeds Diagnosis and Screening Unit, Leeds Institute of Health Sciences, University of Leeds, Leeds.; Cancer Research UK Clinical Trials Unit, Leeds Institute of Clinical Trial Research, University of Leeds, Leeds; NIHR (Leeds) Medtech & In Vitro Diagnostics Cooperative, Leeds.; Cancer Research UK Clinical Trials Unit, Leeds Institute of Clinical Trial Research, University of Leeds, Leeds; NIHR (Leeds) Medtech & In Vitro Diagnostics Cooperative, Leeds.; Leeds Diagnosis and Screening Unit, Leeds Institute of Health Sciences, University of Leeds, Leeds; NIHR (Leeds) Medtech & In Vitro Diagnostics Cooperative, Leeds.; University of Exeter, Exeter.

Multiple myeloma, the third most common blood cancer, is often discovered following multiple prediagnostic consultations, with delays in diagnosis resulting from the ubiquitous nature of presenting symptoms.^1^ Delays result in high disease burden, greater end-organ damage, and emergency presentation, all of which are associated with poorer outcomes.^2^ Improving the early diagnosis of myeloma remains an area of unmet clinical need. In this editorial, we describe the issues with the current diagnostic process, explore the potential impact of the COVID-19 pandemic, and identify alternative strategies that may improve the early diagnosis of myeloma.

## DIAGNOSTIC PROBLEM

Myeloma has one of the longest diagnostic intervals of all cancers. Around half of myeloma patients have three or more pre-referral consultations and around one-third are diagnosed through emergency presentation.^1^^,^^2^ There are various reasons for this. It is a relatively rare cancer; an average GP will see one new case every 8–10 years. Patients present with a range of non-specific symptoms, including back pain, bone pain, fatigue, and blood test abnormalities (hypercalcaemia, renal impairment, anaemia, and raised CRP), which are all common in an ageing population and often attributable to concurrent conditions. Early diagnosis of myeloma is crucial in limiting disease-related complications, such as lytic bone disease, pathological fractures, kidney injury, and severe infection, all of which may impact long-term quality of life, eligibility for routine or experimental therapy, treatment tolerance, response rates, and survival.

## IMPACT OF COVID-19 AND CHANGES TO GENERAL PRACTICE CONSULTATIONS

The COVID-19 pandemic resulted in a substantial decrease in myeloma referrals and diagnoses, in parallel with an increase in emergency presentations.^3^ Reductions in overall urgent referrals for suspected cancer were mainly due to changes in patient attendance, rather than GP referral behaviour. Consultation rates for specific symptoms for other cancers, including red-flag symptoms (such as breast lump and rectal bleeding), reduced during the first lockdown and returned to expected rates within months. However, consultation rates for non-specific symptoms common in myeloma patients (such as back pain, bone pain, and fatigue) remained below expected levels at the end of 2020.^4^ Given the current workload pressures in general practice, patients with non-specific symptoms may prioritise other health concerns, or fail to present at all due to concerns about wasting doctors’ time. GPs have adopted alternative approaches to triage and consulting, including electronic data capture to triage patients and alternatives to face-to-face consultations. GPs may not prioritise non-specific symptoms, as e-consults will automatically ‘red flag’ some symptoms but not non-specific symptoms. We need to understand how this affects patients’ behaviour with non-specific cancer symptoms and presentation in general practice, especially for older patients and those with lower health literacy.

## POTENTIAL OVERDIAGNOSIS OF MGUS

Strategies to facilitate the early diagnosis of myeloma must consider the precursor condition monoclonal gammopathy of undetermined significance (MGUS), often diagnosed incidentally via routine blood tests. MGUS is common, with a prevalence of 3–5% and a risk of progression to myeloma of approximately 1% per year.^5^ Once diagnosed, regular monitoring of blood tests and clinical symptoms is recommended, often guided by MGUS risk-stratification strategies. This has cost and workload implications for the NHS, as well as for the mental health of these patients, the majority of whom will never require treatment for MGUS. Any screening programme for myeloma is likely to increase diagnoses of MGUS and increase the pressures outlined above. A population-based screening trial for MGUS is ongoing in Iceland, where participants with MGUS are randomised to different follow-up strategies.^6^ The results from this will provide vital evidence on the cost-effectiveness of screening and monitoring approaches.

## ALTERNATIVE DIAGNOSTIC STRATEGIES

Many myeloma patients have complex diagnostic journeys and may be seen by other specialists before haematology, potentially delaying diagnosis.^7^ A fundamental challenge is prompting the clinician to consider myeloma and initiate appropriate investigations. Once myeloma is suspected, the diagnostic workup is generally straightforward. Serum protein electrophoresis, immunofixation, and serum free light chain assays are sensitive and specific initial tests for the majority of patients, followed by bone marrow biopsy. Therefore, it is essential that high-risk individuals are identified and appropriately investigated. Strategies to do this with minimal impact on existing NHS resources are needed, particularly in the current COVID-19-recovery phase.

Symptom awareness for both patients and GPs is important to ensure prompt presentation and appropriate referrals. The roll-out of rapid diagnostic centres (RDCs) may provide more timely diagnosis for those with non-specific symptoms by offering a single point of access for further diagnostic investigations, limiting instances of multiple specialty referrals for patients meeting the criteria for multiple site-specific pathways. Evidence emerging from RDC evaluations have shown that around 13% of cancers diagnosed via RDCs are haematological cancers.^8^

Over recent years there has been a major increase in the development of clinical risk prediction models (or algorithms) based on electronic health records. Specifically for myeloma, clinical prediction models developed from general practice records have combined symptom data and blood test results to identify patients at higher risk of developing myeloma.^9^^,^^10^ There is potential to implement such algorithms into decision support tools within GP electronic systems, but challenges remain before these can be fully integrated, such as how this would trigger alerts prospectively and how these would fit with current GP workflows.^11^

Changes in routine blood parameters can be detected several years prior to myeloma diagnosis, before the onset of symptoms or disease complications.^12^ Algorithms based on minor abnormalities and subtle changes in routine blood parameters (such as haemoglobin, liver function tests, and inflammatory markers) may be implemented in hospital laboratories to trigger automatic reflex testing of high-risk individuals for myeloma. This would enable large-scale, low-cost case finding for myeloma, independent of the patient’s ability to articulate their symptoms and the GP considering myeloma as part of the differential diagnosis. Setting appropriate thresholds for testing are challenging and there are legitimate concerns about risk communication, with further research required to explore the acceptability of this approach.

Cell-free DNA (cfDNA) technologies are potentially paradigm shifting for cancer screening. These tests identify circulating tumour DNA and are often able to identify multiple tumour types with a single blood test. The most widely publicised example of this is the methylation-based Galleri® Test, used as part of the NHS-Galleri [GRAIL] study that is exploring population-level cancer screening in asymptomatic individuals (https://www.nhs-galleri.org/). This test can identify plasma cell disorders such as myeloma; however, myeloma and MGUS are genetically very similar and it is not yet clear how well the Galleri test differentiates between the two conditions. There therefore remains a risk of overdiagnosis of low-risk MGUS, the consequences of which may be significant and will only be borne out by prospective studies.

## CONCLUSIONS

Improving the timeliness of myeloma diagnosis is vital to improving patient outcomes, but is difficult to achieve because of complex, non-specific, and varied presentations. Improving GP education on the salient features of multiple myeloma presentation and the investigations required for diagnosis, alongside ensuring adequate safety netting for patients with persistent, unexplained symptoms, should be urgent priorities. Changes to general practice consultations following the COVID-19 pandemic have made myeloma diagnosis more difficult, and, over the longer term, research is required to develop intelligent and technological strategies that support physician decision making and reduce diagnostic delay. These approaches must minimise low-risk MGUS diagnosis, demonstrate economic viability, and have prospective evidence of improvements in objective parameters such as diagnostic speed, disease stage at diagnosis, quality of life, and ultimately survival.

## References

[1] Lyratzopoulos G, Neal RD, Barbiere JM (2012). Variation in number of general practitioner consultations before hospital referral for cancer: findings from the 2010 National Cancer Patient Experience Survey in England. Lancet Oncol.

[2] Howell D, Smith A, Appleton S (2017). Multiple myeloma: routes to diagnosis, clinical characteristics and survival — findings from a UK population-based study. Br J Haematol.

[3] National Disease Registration Service (2022). COVID-19 rapid cancer registration and treatment data. https://www.cancerdata.nhs.uk/.

[4] Nicholson BD, Ordonez-Mena JM, Lay-Flurrie S (2022). Consultations for clinical features of possible cancer and associated urgent referrals before and during the COVID-19 pandemic: an observational cohort study from English primary care. Br J Cancer.

[5] Wadhera RK, Rajkumar SV (2010). Prevalence of monoclonal gammopathy of undetermined significance: a systematic review. Mayo Clin Proc.

[6] Rognvaldsson S, Love TJ, Thorsteinsdottir S (2021). Iceland screens, treats, or prevents multiple myeloma (iStopMM): a population-based screening study for monoclonal gammopathy of undetermined significance and randomized controlled trial of follow-up strategies. Blood Cancer J.

[7] Howell DA, Hart RI, Smith AG (2018). Myeloma: patient accounts of their pathways to diagnosis. Plos One.

[8] Dolly SO, Jones G, Allchorne P (2021). The effectiveness of the Guy’s Rapid Diagnostic Clinic (RDC) in detecting cancer and serious conditions in vague symptom patients. Br J Cancer.

[9] Koshiaris C, Van den Bruel A, Nicholson BD (2021). Clinical prediction tools to identify patients at highest risk of myeloma in primary care: a retrospective open cohort study. Br J Gen Pract.

[10] Shephard EA, Neal RD, Rose P (2015). Quantifying the risk of multiple myeloma from symptoms reported in primary care patients: a large case–control study using electronic records. Br J Gen Pract..

[11] Bradley PT, Hall N, Maniatopoulos G (2021). Factors shaping the implementation and use of Clinical Cancer Decision Tools by GPs in primary care: a qualitative framework synthesis. BMJ Open.

[12] Koshiaris C, Van den Bruel A, Oke JL (2018). Early detection of multiple myeloma in primary care using blood tests: a case–control study in primary care. Br J Gen Pract.

